# The Construction of Multi-Incorporated Polylactic Composite Nanofibrous Scaffold for the Potential Applications in Bone Tissue Regeneration

**DOI:** 10.3390/nano11092402

**Published:** 2021-09-15

**Authors:** Du Nie, Yi Luo, Guang Li, Junhong Jin, Shenglin Yang, Suying Li, Yu Zhang, Jiamu Dai, Rong Liu, Wei Zhang

**Affiliations:** 1 Key Laboratory of Neuroregeneration of Jiangsu and Ministry of Education, Co-Innovation Center of Neuroregeneration, School of Textile and Clothing, Nantong University, Nantong 226001, China; flory2114@hotmail.com (D.N.); lynantong@hotmail.com (Y.L.); lisy@ntu.edu.cn (S.L.); z.yu@ntu.edu.cn (Y.Z.); 2 State Key Laboratory for Modification of Chemical Fibers and Polymer Materials, College of Materials Science and Engineering, Donghua University, Shanghai 201620, China; lig@dhu.edu.cn (G.L.); jhkim@dhu.edu.cn (J.J.); slyang@dhu.edu.cn (S.Y.)

**Keywords:** bone tissue engineering, nanofibrous scaffold, polysilsesquioxane, pearl powder, dexamethasone, porous carbon nanofibers

## Abstract

To improve the bone regeneration ability of pure polymer, varieties of bioactive components were incorporated to a biomolecular scaffold with different structures. In this study, polysilsesquioxane (POSS), pearl powder and dexamethasone loaded porous carbon nanofibers (DEX@PCNFs) were incorporated into polylactic (PLA) nanofibrous scaffold via electrospinning for the application of bone tissue regeneration. The morphology observation showed that the nanofibers were well formed through electrospinning process. The mineralization test of incubation in simulated body fluid (SBF) revealed that POSS incorporated scaffold obtained faster hydroxyapatite depositing ability than pristine PLA nanofibers. Importantly, benefitting from the bioactive components of pearl powder like bone morphogenetic protein (BMP), bone mesenchymal stem cells (BMSCs) cultured on the composite scaffold presented higher proliferation rate. In addition, by further incorporating with DEX@PCNFs, the alkaline phosphatase (ALP) level and calcium deposition were a little higher based on pearl powder. Consequently, the novel POSS, pearl powder and DEX@PCNFs multi-incorporated PLA nanofibrous scaffold can provide better ability to enhance the biocompatibility and accelerate osteogenic differentiation of BMSCs, which has potential applications in bone tissue regeneration.

## 1. Introduction

Bone tissue engineering has developed to be an essential method for therapy of bone defects caused by mechanical injury, osteoporosis and other diseases [1,2,3]. As a wildly applied strategy to overcome limitations including availability and potential disease transmission from current treating methods of autogenous and allogenous bone grafting, synthetic scaffolds with different components and structures are proposed to provide the necessary support for cell proliferation and mechanical function [4,5,6]. The scaffolds made from biocompatible and biodegradable materials, such as synthetic polymers of polylactic (PLA) and natural silk fiber (SF), are getting more attention by researchers for the benefit of leaving room to the new bone tissue [7,8,9,10]. As a versatile technique, electrospinning is used to prepare nanofibers based on varieties of materials for the application of water treatment, filtration, drug loading and catalysis. In addition, the structure of nanofibers is able to mimic the extracellular matrix (ECM) to support cell adhesion and proliferation, which has been widely considered to be excellent in tissue regeneration including bone, vascular, skin and nerve conduit [11,12,13,14,15]. However, due to the weak bioactivity of single component of pure polymer, some bioactive additives are attempted to incorporate with the polymer scaffolds to enhance the corresponding capacity of accelerating tissue regeneration.

Some growth factors will attend the regulation of bone formation process, such as bone morphogenetic protein (BMP), transforming growth factor β (TGF-β) and insulin like growth factor (IGF) [16,17,18]. Particularly, as a recombinant bone morphogenetic protein, BMP-2 is introduced to scaffolds by surface functionalization or direct incorporation to stimulate bone regeneration, whereas due to the problems that dissociative BMP-2 will be degraded rapidly by proteinases in the body and high dosage will bring some adverse effects including immunological reaction and abnormal bone formation, BMP-2 immobilization onto the scaffold is a suitable method to improve the activity and utilization [19,20,21,22]. Pearl powders have been found to be natural composites of inorganic CaCO_3_ and organic matters including BMP-2, which makes it show better bone regeneration activity than hydroxyapatite (HA), the main inorganic component of natural bone [23,24,25,26,27]. In addition, compared to the method of chemical modification, a BMP-2 immobilization structure is naturally constructed without any other process, which can be directly used in bone regeneration avoiding the concerns mentioned above.

To further enhance the capacity of bone regeneration, some drugs have been introduced with growth factors to perform a combined therapy, for instance, dexamethasone (DEX) is proven to be an osteogenic inducer to bone marrow stromal cells (BMSCs) [28,29,30,31]. Similarly, a high dosage of DEX will also bring some adverse effects and flow away quickly with body fluid. Thus, a drug carrier has been applied for DEX loading and controlled release [28,32,33,34].

Moreover, except for the stimulation of cell activity, calcium deposition is another crucial property to new bone formation. Generally, pure polymer cannot quickly deposit calcium unless incorporated with some additives, which can play a role as nucleus or obtain special groups to catch calcium. Silica based bioceramics have been proven to chemically combine with bone tissue and promote new bone healing and growth [35,36,37]. Polyhedral oligosilsesquioxane (POSS), a well-designed cage structural molecule with various functional groups outside, is proposed as a great candidate to improve HA crystal formation [38,39,40].

In this study, a multi incorporated composite PLA nanofibrous scaffold was prepared with POSS, DEX and pearl powder by electrospinning, and the controlled release of DEX was realized by dexamethasone loaded porous carbon nanofibers (DEX@PCNFs). The effects of these components on mineralization, cytotoxicity and osteogenic differentiation of BMSCs were investigated to evaluate the potential application in bone tissue engineering.

## 2. Materials and Methods

### 2.1. Fabrication of Polysilsesquioxane-Blended PLA Nanofibers

The polysilsesquioxane (POSS, AM0275, Hybrid Plastics, Inc., Hattiesburg, MS, USA) incorporated polylactic (PLA, WM = 2 × 10^6^, Guanghua Weiye Co., Ltd., Shenzhen, China) nanofibers were fabricated by electrospinning as reported in our previous work [41]. Briefly, a hybrid solution (N,N-dimethylformamide (DMF), dichloromethane (DCM) and hexafluoroisopropanol (HFIP), the volume ratio is 1:3:3) was used to dissolve PLA (10 wt%) and POSS (weight ratio to PLA is 0 wt%, 2 wt%, 4 wt% and 6 wt%), and the solution was fed into plastic syringe for electrospinning under conditions including voltage of 18 kV, extrusion rate of 0.9 mL/h and collecting distance of 18 cm between needle tip and aluminum foil. The obtained samples were placed in 60 °C vacuum oven to completely remove solvent. For pristine PLA, the nanofiber sample was marked as PLA-NF, and POSS incorporated scaffolds were marked as PS-NF, for instance, PS2-NF mean the POSS is 2 wt% to PLA. Afterwards, all the samples were observed by scanning electron microscope (SEM, JSM-5600 LV, JEOL, Tokyo, Japan) to analyze the influence of POSS amount on the nanofiber morphology and determine the suitable sample for the following experiment. In addition, the nanofibrous mets were cut into 4 cm × 1 cm to evaluate the mechanical properties by electronic universal testing machine (Instron 5969, Norwood, MA, USA) with a cross-head speed of 5 mm/min.

### 2.2. Mineralization Test

To measure the HA deposition ability, the scaffolds were incubated in simulated body fluid (SBF) prepared as the reference [42] for a certain period at 37 °C. During the incubation period, SBF would be changed each day. Then, the samples were moved out, washed with deionized water and dried in 60 °C vacuum oven for observation of surface change and analysis of POSS influence on HA deposition.

### 2.3. Preparation of Dexamethasone Loaded Porous Carbon Nanofibers

Firstly, porous carbon nanofibers (PCNFs) were prepared from polyacrylonitrile (PAN) and polymethylmethacrylate (PMMA) via electrospinning with the same procedures and factors as Part 2.1, followed by pre-oxidation 280 °C for 2 h and carbonization under 800 °C for 2 h of PAN/PMMA blend nanofibers. Then, a mixed acid solution of H_2_SO_4_ and HNO_3_ (volume ratio is 3:1) was used to treat the surface of PCNFs under 70 °C to make it hydrophilic and able to disperse in a water environment. For drug loading, 1 g dexamethasone (DEX) and 1 g PCNFs were added in 10 mL deionized water; the solution was ultrasonicated and kept stirring for 24 h under dark condition. Afterwards, the solution was centrifuged, and the solid was washed with deionized water to obtain DEX@PCNFs. The supernatant and washing solution were collected to determine the DEX loading amount by using UV–VIS spectra at wavelength of 242 nm. Before calculating the loading efficacy of DEX on PCNFs, a DEX standard curve of adsorption and concentration was studied. Subsequently, DEX solutions before and after adsorption were scanned by UV–VIS to obtain adsorption value, and corresponding concentrations were calculated according to the standard curve. Finally, the mass change, loading amount was further calculated.

### 2.4. Fabrication of Multi-Incorporated Scaffold

Based on the fabrication of PS-NF in Part 2.1, pearl powder (average diameter of 100 nm, Guanghua Weiye Co., Ltd., Shenzhen, China) and DEX@PCNFs were further added in the electrospinning to prepare multi-incorporated nanofibrous scaffold. To guarantee the success of electrospinning process, the adding amount of DEX@PCNFs was 1 wt% to PLA, and pearl powder was 5–15 wt%. Due to the amount of POSS and DEX@PCNFs was determined, the as-prepared multi-incorporated scaffold was marked as PSCP-NF according to the variation of pearl powder amount, for instance, PSCP5-NF mean that pearl powder is 5 wt% to PLA. The morphology and distribution of pearl powder and DEX@PCNFs were observed by SEM and transmission electron microscope (TEM, JEM-2100, JEOL, Tokyo, Japan).

### 2.5. Cell Culture

Bone mesenchymal stem cells (BMSCs, Chinese Academy of Science, Shanghai, China) of fourth to sixth generation were selected to complete all the in vitro experiments in this work. The cells were cultured in normal complete medium of low glucose DMEM containing 10% FBS and 1% penicillin/streptomycin at 37 °C and 5% CO_2_ for normal proliferation cytotoxicity assay. When it came to the osteoconducting test, the normal medium was supplemented with 10 mM β-glycerol phosphate and 50 μg/mL of L-ascorbic acid. During all the culture period, the medium would be replaced every 3 days.

### 2.6. Cytotoxic Assay

The cytotoxicity of different samples was measured by MTT assay. Briefly, BMSCs were seeded on the surface of sanitized samples in 24-well plate with 400 μL normal complete medium at a density of 2 × 10^4^ cells per well. After incubating for pre-determined time point, the samples were washed with PBS and continued to be incubated with 360 μL fresh low glucose DMEM and 40 μL MTT solution (5 mg/mL) per well for another 4 h. Then, the solution was removed, and 400 μL dimethylsulfoxide (DMSO) per well was added for further 0.5 h incubation in a 37 °C shaker for 10 min under dark condition. A volume of 100 μL of the supernatant per well was transferred to 96-well plate for the absorbance measurement at wavelength of 570 nm with a microplate reader (Multiskan GO, Thermo Fisher, Waltham, MA, USA). Every scaffold would set at least 3 parallel samples, and the average and standard deviation (SD) of the absorbance data were analyzed.

For cell adhesion evaluation via confocal laser scanning microscopy (CLSM), BMSCs were seeded on the scaffolds and cultured for 3 days, then washed with PBS and fixed with 4% glutaraldehyde for 30 min at 4 °C. Afterwards, the cells were washed with PBS and soaked in 0.1% Triton X-100 solution for 5 min. After washing with PBS again, the cells were stained in turn with Alexa Flour@ 488 phalloidin solution (165 nM) and DAPI solution (100 nM) to label cytoplasm and nucleus, respectively. Finally, the samples were washed with PBS for CLSM observing.

### 2.7. Alkaline Phosphate Activity Test

The alkaline phosphate (ALP) activity of BMSCs was tested by corresponding assay kit (Beyotime Institute of Biotechnology, Shanghai, China). Briefly, after incubating with different samples in conducting medium at pre-determined time points, BMSCs were lysed by cell lysis buffer, and 50 μL cell lysate was transferred into 96-well plate with 50 μL substrate solution for 30 min incubation at 37 °C, followed by adding 100 μL stop solution to terminate the reaction for evaluating the absorbance at wavelength of 405 nm via plate reader. In addition, another 10 μL cell lysate was used to measure the total protein concentration by protein assay kit (Beyotime Institute of Biotechnology, Shanghai, China).

### 2.8. Alizarin Red S Staining

After incubating in conducting medium for pre-determined time point, BMSCs were fixed with 4% glutaraldehyde for 30 min and washed with PBS. Deionized water was further used to wash the samples to remove residual salt for the purpose of staining BMSCs with 2% (*w*/*v*) alizarin red S (ARS) solution with adjusted pH value of 4.1–4.3 for 10 min at room temperature. Then, ARS solution was removed, the sample was washed with deionized water until there was no residual ARS to obtain the stained sample, which was photographed with a camera.

### 2.9. Statistical Analysis

Statistical analysis was carried out through a one-way analysis of variance (one-way ANOVA) and Scheffe’s post hoc test. The statistical significance for all tests were considered at * *p* < 0.05 and ** *p* < 0.01 [43,44].

## 3. Results and Discussion

### 3.1. Fabrication and Characterization of PS-NF

**Figure 1:**
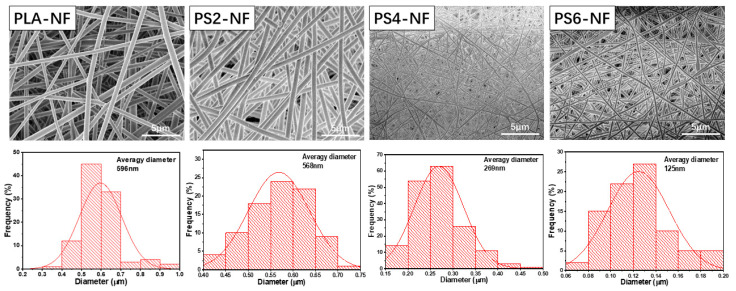
SEM images and diameter distribution of PLA NF with different POSS ratio.

To improve the mineralization ability of PLA nanofibrous scaffold, POSS was added with different amount. The surface of as-prepared nanofibers was observed with SEM and showed in Figure 1. As we can see, PLA-NF scaffold was well prepared with smooth surface, the average diameter was calculated to be around 596 nm, and PS2-NF presented similar morphology as PLA-NF with average diameter of about 568 nm, whereas with the increase of POSS amount, the nanofiber diameter greatly decreased, and some adhesion areas were generated among the fibers. In addition, the mechanical properties showed that PLA-NF and PS2-NF had similar tensile strength, while that of scaffold with higher POSS amount was much lower (Table 1), even though PS6-NF cannot be tested. These results might contribute to the plasticizer effect of POSS decreasing the viscosity of polymer solution [45], which decreases the diameter of nanofibers during electrospinning process. The much lighter weight of single nanofiber also made the scaffold fluffier, which resulted in the bigger porosity and lower mechanical property. Therefore, the POSS amount was determined to be 2 wt% to PLA, which was used in the following experiments.

**Figure 2:**
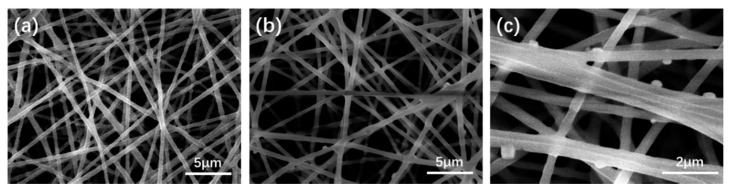
SEM images of (**a**) PLA-NF and (**b**) PS2-NF after mineralization for 3 days, (**c**) is high magnification of (**b**).

Mineralization property is an important aspect of the scaffold for the application of bone regeneration field. In this study, HA depositing capacity of the scaffold was investigated to reveal the effect of POSS incorporation. After incubating with SBF for 3 days, the morphology change of PLA-NF and PS2-NF was observed with SEM to find deposited HA particles. As shown in Figure 2, no obvious change was found on PLA-NF, the surface remained smooth, while some small particles were adhered on the surface of PS2-NF, indicating that the incorporation of POSS could successfully capture Ca^2+^ from SBF accelerate HA deposition [38].

### 3.2. Preparation of DEX@PCNFs

**Figure 3:**
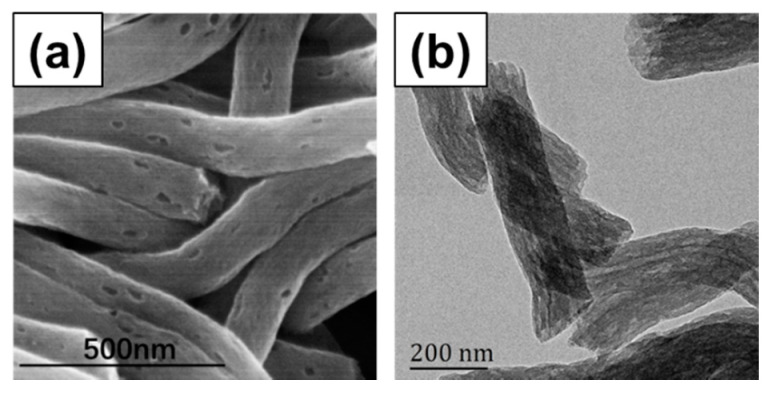
(**a**) SEM and (**b**) TEM images of PCNFs.

PCNFs were applied as drug carriers in this study, which were prepared through electrospinning of PAN and PMMA, followed by pre-oxidation and carbonization. As reported in our previous work, due to the different physical and chemical behavior of these two polymers during the preparing process, PAN would transform into carbon framework, while PMMA would be decomposed into H_2_O and CO_2_ [46]. As shown in Figure 3, the mesopores, which were beneficial to improve the drug loading amount, were well formed on the surface and the axial channel of the inside. After blending with DEX solution, DEX was successfully loaded in PCNFs to obtain a drug-loading system DEX@PCNFs, and the loading amount was measured to be 53.4 mg/g through UV–VIS spectra.

### 3.3. Fabrication and Characterization of PSCP-NF

**Figure 4:**
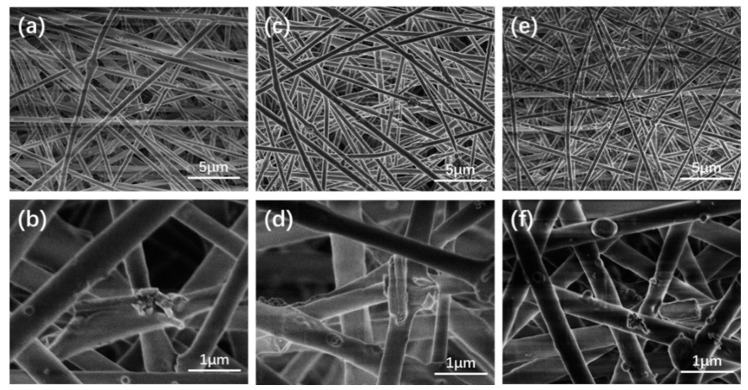
SEM images of (**a**,**b**) PSCP5-NF, (**c**,**d**) PSCP10-NF and (**e**,**f**) PSCP15-NF; TEM images of PSCP5-NF.

**Figure 5:**
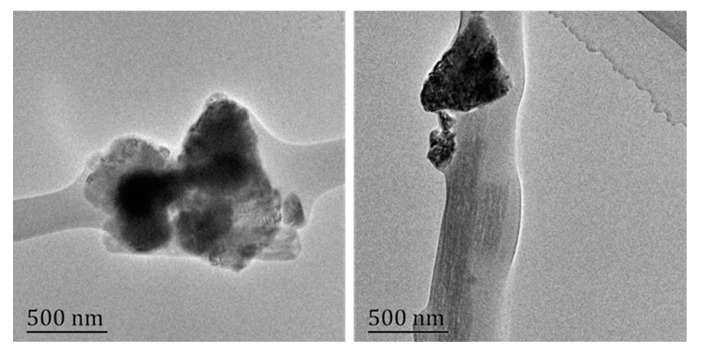
TEM images of PSCP5-NF.

Besides the applied POSS and DEX@PCNFs, pearl powder was also incorporated in the nanofibrous scaffold. As shown in Figure 4, the SEM pictures of scaffold with pearl powder concentration of 5 wt%, 10 wt% and 15 wt% to PLA, as well as 2 wt% POSS and 1 wt% DEX@PCNFs, illustrated that the pearl powder and PCNFs could be distributed on the surface of the nanofibers without obvious influence on the nanofiber forming during the electrospinning process. In addition, the TEM pictures of PSCP5-NF (Figure 5) showed that pearl powder and PCNFs could also be wrapped inside the PLA nanofiber. Moreover, no fiber breakages were found due to the blending of these additives, despite the morphology of PLA-NF becoming a little rough to some degree. Benefitting the coverage of PLA shell, the release rate of DEX would further slowdown based on the controlled release from PCNFs, which would decrease the waste of DEX along with body fluid flowing and prolonging the working period of DEX in the cells.

### 3.4. Cytotoxicity of PSCP-NF

**Figure 6:**
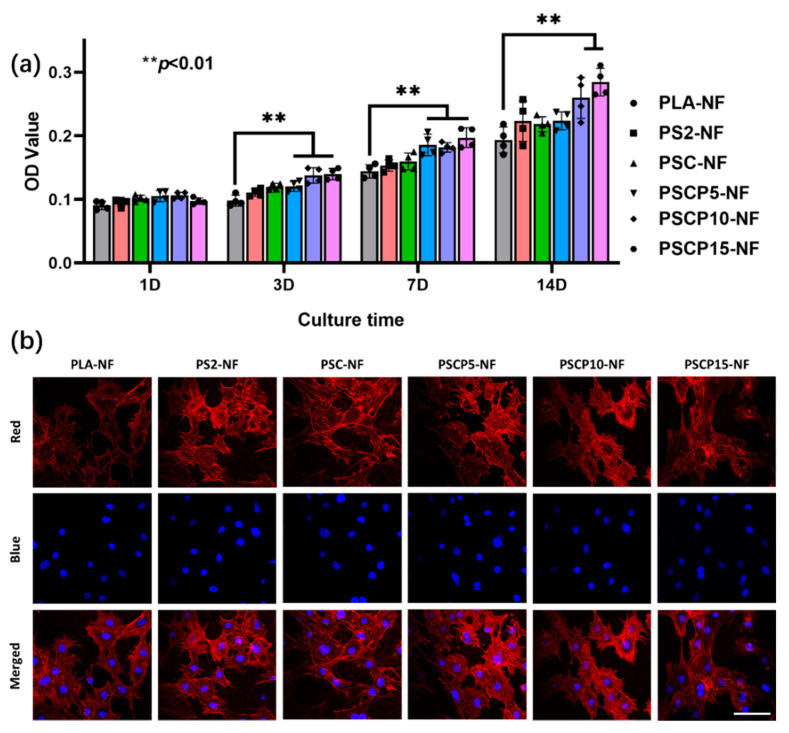
(**a**) MTT assay of various samples after incubation with BMSCs for different time points; (**b**) confocal laser scanning microscopy images of BMSCs seeded on various scaffolds after incubating for 3 days; the red presents the cytoplasm, and the blue presents the nucleus. Scale bar = 75 μm.

The investigation of the cytotoxicity of the as-prepared scaffold should be carried out before the cell differentiation test. BMSCs were applied to this in vitro experiment with MTT assay. The O.D. value of various scaffolds was presented in Figure 6a; it can be found that all the composite scaffolds showed no obvious cytotoxicity on BMSCs when comparing with pristine PLA-NF during the 2 weeks incubating period. In addition, the proliferation rate was gradually raised with the increase of additive amount, especially in long-time incubation. As expected, with the increase of the amount of pearl powder, the scaffold showed an increasing tendency in proliferation rate. In addition, the morphology of BMSCs was observed by CLSM and presented as Figure 6b; all cells on different scaffold showed stretched morphology with pseudopodium along the nanofiber axial direction, which suggested that the additives would not affect the adhering and cloning behavior. The results indicated that multi-incorporated scaffold is much biocompatible and suitable for the following experiments.

### 3.5. ALP Activity Test

**Figure 7:**
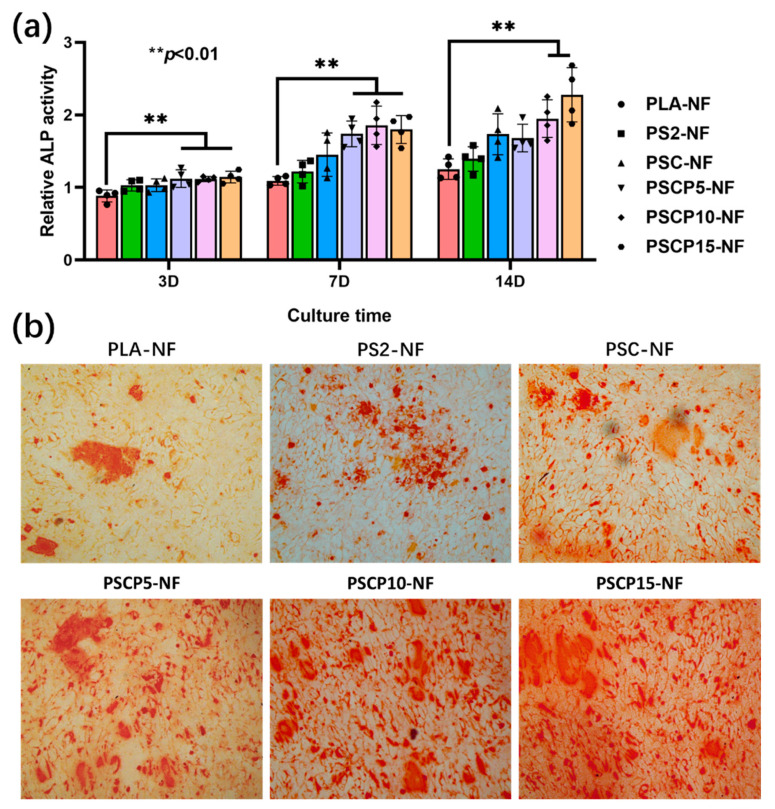
(**a**) ALP activity of BMSCs after incubating with various samples for different time points (signification difference compared to other groups ** *p* < 0.01); (**b**) digital photos of ARS-stained mineralized matrix on various samples after osteogenic incubation for 14 days.

In this study, POSS was applied to accelerate HA deposition, which is the main inorganic component of natural bone tissue and could raise osteoconduction ability to BMSCs. DEX, a bioactive agent to improve the osteogenic differentiation activity of BMSCs, was loaded on PCNFs to construct drug-controlled release system. Pearl powder in nanosize was also used in bone tissue regeneration due to the bioactive component including BMP-2, which is a peptide benefiting to enhance BMSCs differentiation. Based on the above advantages of these materials, the multi-incorporated scaffold was expected to obtain much better osteoblastic differentiation activity comparing with pristine PLA. As a typical mark of differentiation, ALP activity of BMSCs incubated with different scaffold was measured, the upregulation of which is a key signal at the early period of bone regeneration. The results were showed in Figure 7a, all composite scaffolds obtained higher ALP activity than pristine PLA, despite PLA being a great biocompatible polymer, while hardly being able to present bioactivity to support cell differentiation, whereas the released DEX from PCNFs and BMP-2 like bioactive components from pearl powder would well improve the ALP activity of BMSCs after osteoconducting incubation for 2 weeks. Obviously, with the increase of pearl powder amount in the scaffold, the ALP activity would also be upregulated. Therefore, the multi-incorporated nanofibrous scaffold showed great ability to enhance ALP activity of BMSCs.

### 3.6. In Vitro Mineralization

BMSCs would synthesize calcium deposition as mineralized product during the osteoconducting incubation period, which could be stained to red with ARS solution. After 2 weeks of differentiation, the ARS-stained samples were observed under optical microscopy and presented in Figure 7b, the calcium deposition was well formed on the scaffolds. Due to the low osteoconductivity of PLA, the area of calcium deposition was not very big. However, after incorporating with POSS, DEX@PCNFs and pearl powder, this area was obviously bigger than PLA, which indicated that ability of calcium deposition synthesis was remarkably improved. According to the above results, the multi-incorporated scaffold could exert all the positive effect on the osteogenic differentiation of BMSCs.

## 4. Conclusions

In summary, a multi-incorporated PLA nanofibrous scaffold with different functional components was prepared through electrospinning for the application of bone tissue engineering. The mineralization result showed the POSS in the scaffold could accelerate HA deposition, which is beneficial to reconstruct bone tissue structure. Moreover, PCNFs were prepared and used to load DEX to obtained drug delivery system of DEX@PCNFs, which was co-incorporated with pearl powder to the scaffold. The in vitro experiment demonstrated that the DEX and bioactive component from pearl powder would contribute to the improving of biocompatibility, upregulating ALP activity and increasing calcium deposition of BMSCs based on pure PLA. Therefore, the multi-incorporated PLA nanofibrous scaffold obtained better osteogenic differentiation ability and showed bright potential for bone tissue regeneration.

## Figures and Tables

**Table 1:** Mechanical properties of PLA NF with different POSS ratio.

	PLA-NF	PS2-NF	PS4-NF	PS6-NF
Tensile strength (MPa)	6.7 ± 0.3	6.1 ± 0.5	1.2 ± 0.5	-
Elongation at break (%)	86.8 ± 4.5	95.3 ± 6.2	41.8 ± 8.6	-

## Data Availability

The data used to support the findings of this study are available from the corresponding author on request.
